# Pregnant Women’s Awareness of Periodontal Disease Effects: A Cross-Sectional Questionnaire Study in Saudi Arabia

**DOI:** 10.3390/healthcare12232413

**Published:** 2024-12-01

**Authors:** Khalid Saad Alkhurayji, Arwa Althumairi, Abdulmunim Alsuhaimi, Sultan Aldakhil, Abdulrahman Alshalawi, Muath Alzamil, Farah Asa’ad

**Affiliations:** 1 Health Information Management and Technology Department, College of Public Health, Imam Abdulrahman bin Faisal University, Dammam 34212, Saudi Arabia; aalthumairi@iau.edu.sa; 2 Executive Department of Standards, Saudi Central Board for Accreditation of Healthcare Institutions, Riyadh 12264, Saudi Arabia; aalsuhaimi@cbahi.gov.sa; 3 Restorative and Prosthetic Dental Sciences Department, College of Dentistry, King Saud Bin Abdulaziz University for Health Sciences, Riyadh 11481, Saudi Arabia; dakhils@ksau-hs.edu.sa; 4 King Abdullah International Medical Research Center, Ministry of National Guard Health Affairs, Riyadh 11481, Saudi Arabia; 5 Dental Health Department, College of Medical Applied Sciences, King Saud University, Riyadh 12372, Saudi Arabia; 441100546@student.ksu.edu.sa; 6 Department of Dental Health Care, College of Applied Medical Sciences, Inaya Medical Colleges, Riyadh 13541, Saudi Arabia; malzamil@inaya.edu.sa; 7 Department of Oral Biochemistry, Institute of Odontology, Sahlgrenska Academy, University of Gothenburg, 405 30 Gothenburg, Sweden; farah.asaad@gu.se

**Keywords:** oral health, awareness, pregnant women, women’s health, health education, questionnaire

## Abstract

Background/Objectives: Periodontal disease is an infection of the surrounding and supporting tissues of the teeth. Several associations have been identified for systemic conditions. This study aimed to assess the awareness of pregnant women regarding the link between premature and low-weight births with periodontal diseases. Methods: Using a cross-sectional design, we collected data from the target participants through the Pregnant Women’s Periodontal Awareness Questionnaire, employing a convenience sampling technique between November and December 2023. Statistical analyses were performed using descriptive statistics. The statistical significance of all estimations was set at a *p*-value of ≤0.05. Results: Most participants were ≥31 years of age (210 participants; 67.3%). The percentage of pregnancies occurring in women who were multigravida was 69.6%. Only 29.5% of the participants were knowledgeable about low-weight and premature births with periodontal diseases. The findings indicate an association between the education level and trimester (*p* < 0.05). Conclusions: Pregnant women’s awareness regarding periodontal disease is low, and one-third of them do not visit dental clinics during pregnancy. Expanding access to dental care and reducing anxiety about treatment outcomes can strengthen the relationship between oral health and general health.

## 1. Introduction

Periodontal disease is an infection of the surrounding and supporting tissues of the teeth, mostly caused by bacterial plaque accumulation [1]. Gum inflammation begins with gingivitis and can advance to periodontitis, which is characterized by the breakdown of hard tissue involving the periodontal ligament and bone [2]. Bacteria like Prophyromonas gingivitis drive this disease, as does the host immune response, which produces cytokines that lead to tissue and bone loss [3]. Several risk factors can influence periodontal disease, including smoking, hormonal changes, diabetes, and genetics [4,5]. Several associations have been identified for systemic conditions such as cardiovascular disease, diabetes, and pregnancy outcome complications, including low birth weights and early births [6,7,8]. Signs and symptoms of periodontal disease include bleeding, reddishness, and swollen gums [9]. The main cause of periodontium-related disease is poor oral hygiene. Furthermore, scaling and root planning are used to treat and manage periodontitis. However, several complications may occur if left untreated, such as tooth loss and the entry of bacteria into the bloodstream, which causes systematic complications [10]. Brushing and flossing daily and knowing the risk factors of periodontal disease can help prevent periodontal disease [9].

Severe periodontitis is associated with early births and low birth weights [11]. Inflamed gingiva can allow bacteria to enter the bloodstream, causing inflammatory damage that leads to contractions and premature and low birth weight [12]. Nonetheless, several mechanisms are related to periodontal diseases, which cause adverse pregnancy outcomes such as placental dysfunction, endothelial dysfunction, oxidative stress, and immunological dysregulation [13]. Furthermore, a two-year study involving over 3000 pregnant women in India evaluated the association between periodontitis during pregnancy and low and premature births [14]. In addition, a recent meta-analysis found a moderate association between periodontitis and low birth weight and a weak association between premature birth and periodontitis [15]. Furthermore, according to a systematic review of randomized control trials, the birth outcome among women with periodontitis during pregnancy did not improve by scaling and non-surgical periodontal treatment. However, more rigorous treatment before conception, which targets the inflammation of oral pathogens such as Fusobacterium nucleatum, can minimize the risk of low-weight and premature births [16].

A meta-analysis of randomized controlled trials illustrates that three periodontal treatment intervention techniques reduced the risk of premature and low-weight births. First, sub- and supra-scaling with root planing and chlorhexidine mouthwash. Second, sub- and supra-scaling with root planing, chlorhexidine mouthwash, polishing, and plaque control. Third, sub- and supra-scaling with root planing and chlorhexidine mouthwash with a sonic toothbrush in addition to tooth polishing and plaque control [17]. Furthermore, a meta-analysis of the risk of low birth weight and premature birth in women with periodontal diseases found a consistent association. Additionally, the significant risk of premature birth was lower than the risk of low birth weight among women during pregnancy with periodontitis [18].

At present, the worldwide incidence of periodontal disease is estimated to be 1,087,367,744.0 cases, with 91,518,820.6 new cases, which is twice as many as in 1990 [19]. Several risks are related to periodontitis during pregnancy. For instance, age associated with hormonal changes may also be a risk factor for modifying periodontal diseases among women during pregnancy [20]. To illustrate this case, a systematic analysis revealed that women aged 18 to 41 (the age range in which women often become pregnant) are at an increased risk of gingival inflammation, decreased salivary flow, and increased biofilm accumulation during pregnancy. In addition, periodontal disease severity usually worsens and increases with age, reaching its peak in the third trimester [21]. A recent nationwide survey regarding the prevalence of periodontal illnesses in Indonesia indicated that more than 65% of Indonesians experience periodontal illnesses, with the most alarming factors associated with periodontal disorders being lack of education, tooth brushing habits, and smoking [22].

According to the literature, pregnancy can increase the risk of periodontitis, given that progesterone and estrogen have an important role in changing the risk of periodontal diseases [21]. Maternal periodontitis can arise for a variety of reasons, including an increased inflammation burden, dysbiosis of the intrauterine microenvironment, and epigenomic alterations, complicating the diagnosis [23]. Still, oral health during pregnancy is frequently overlooked, and the majority of pregnant women do not visit dental clinics or seek treatment for periodontal diseases [24]. Periodontal disease treatment during pregnancy has previously been shown to be safe and beneficial for oral health. More importantly, it can help reduce the effects of diabetes, pre-eclampsia, fetal growth complications, and cardiovascular diseases in the long term [25]. Despite advancements in periodontal therapy and high expectations for treatment outcomes, the essential management option remains regular scaling and polishing along with plaque control [26].

Awareness levels vary among countries. For instance, in Italy, more than 40% of pregnant women are unaware of the link between periodontal diseases and premature births or low birth weights [27], while more than 65% of Sri Lanka’s population is unaware of periodontal disease [28]. In a different context, 33% of respondents correctly answered a question about the relationship between premature births and low birth weight and periodontal disease in India [29]. In Saudi Arabia, however, in the region of Alahsa, it was discovered that 57% of pregnant women are unaware of the harm that dental illnesses can cause to their unborn child [30].

Multiple factors may restrict maintaining oral health despite existing awareness, indicating a need for an improved understanding of oral health and its treatments [22]. However, increased levels of anxiety may emerge as a result of mental distress, fear, or the perception that dental care is unnecessary [31]. In addition, sensory sensitivity is a physical reaction to stimuli that cause patients to be sensitive to pain and suffering during treatment, particularly dental treatment, and during experiences with oral disorders [32].

The awareness of periodontal diseases in pregnant women is crucial as it can reduce the risk of complications, facilitate early detection and treatment, and encourage better oral hygiene practices [33]. Nonetheless, some researchers suggest that periodontal diseases could impact the quality of life. For instance, previous studies found a substantial link between periodontal diseases and quality of life [34]. Furthermore, Falcao and Bullón [35] identified a relationship between periodontal diseases and systemic illnesses, suggesting a negative association between overall health and oral health.

Raising awareness about the connection between periodontal disease and adverse pregnancy outcomes, particularly low birth weights and premature births, can significantly impact public health. By promoting the understanding of these issues, we can improve health outcomes for pregnant women and their unborn children, as periodontitis can be severe and dangerous throughout pregnancy. Therefore, this study intends to assess the awareness of pregnant women regarding the link between low birth weights and premature births with periodontal diseases at a single secondary hospital.

**H_0_.:**  *Pregnant women during the second trimester are not more likely to visit a dental clinic than those in other trimesters.*

## 2. Materials and Methods

### 2.1. Design of the Study

We used a descriptive quantitative cross-sectional questionnaire-based study to evaluate the awareness of pregnant women regarding the association between premature births and low birth weights and periodontium diseases at a single secondary hospital.

### 2.2. Study Participants

This study included pregnant women who visited the Obstetrics and Gynecology Department during pregnancy at a single secondary hospital within the study period (30 November to 30 December 2023). Eligible participants were those who had access to prenatal care, and dental services were included.

The exclusion criteria were as follows:

a. Pregnant women who were unable to communicate effectively in Arabic or English, as the study materials were only available in these languages.
b. Pregnant women with severe mental illnesses, as this might affect their ability to understand the study’s aim and scope.
c. Pregnant women who declined to provide informed consent.
d. Women who were postpartum or had ectopic pregnancies.

A convenience sampling method was used to collect responses from participants in the pregnancy waiting area. Data collectors directly handed out paper-based questionnaires to the participants, who were instructed to fill them out accordingly.

### 2.3. Ethical Considerations

Approval from the ethical review committee was granted before the beginning of the study by the Institutional Review Board (or Ethics Committee) of Prince Sultan Military Medical City (ref. no.: E-2213 on 9 November 2023). Informed consent, an explanation of the research aims, the confidentiality of the identity, results communication, and a statement of voluntary participation were provided on the first page of the questionnaire.

### 2.4. Data Collection

A 16-item questionnaire was distributed to eligible participants in the waiting area. The minimum sample size required was estimated using G-Power 3.1.1 [36]. Calculation of the sample size resulted in a minimum of 143 participants by taking into account an effect size of 0.3 calculated from previous studies [37,38], a study power of 0.8, and an alpha error probability of 0.05.

The pilot study was conducted with 30 participants to evaluate the internal consistency of the questionnaire using Cronbach’s alpha coefficient. Hence, since the adopted tool employed Cronbach’s alpha for the previous study, we used the same measurement to assess the internal consistency of the questionnaire and examine the instrument’s psychometric properties over time [39].

### 2.5. Instrument

This study adopted a paper-based pre-validated questionnaire entitled “Pregnant Women’s Periodontal Awareness Questionnaire”. The study contained 16 items in English assessing awareness of dental health, including periodontal-related information, and the Cronbach’s alpha score was (0.87) [39]. Additionally, the questionnaire was translated into Arabic using backward–forward translation (Supplement S1).

The first part was a cover letter that included the study goals and objectives and an informed consent statement. The second part comprised demographic information, pregnancy details, and awareness using closed-ended questions.

### 2.6. Analysis of Statistics

The pre-analysis phase involved coding the results to prepare for the analysis of the study statistics using IBM SPSS Statistics version 25 [40]. Univariate analyses were performed for descriptive statistics such as frequency and percentage. Bivariate analysis was also performed using the Chi-square test. Binary logistic regression was performed after fulfilling the assumption of the regression model. A *p*-value of ≤0.05 was considered statistically significant for all estimations. The dependent variable in this study was the awareness of pregnant women about periodontal diseases. The independent variables were age, education level, stage of pregnancy, and number of pregnancies.

## 3. Results

**Figure 1:**
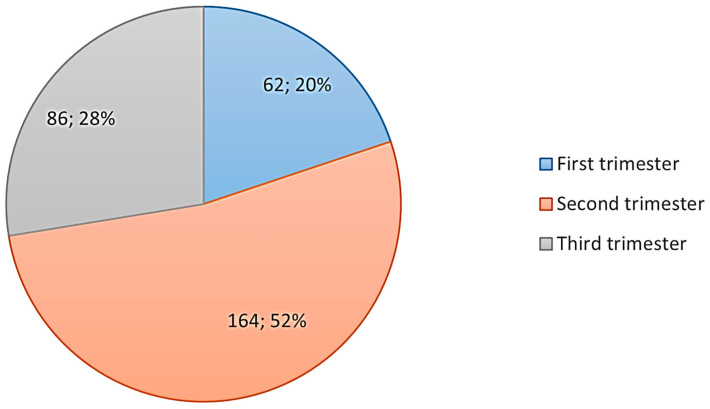
Participants’ stage of pregnancy.

A total of 312 women during pregnancy participated in this study. The majority of the pregnant women were ≥31 years old (210 participants; 67.3%). Only 32.7% of the participants were 30 years old or younger. More than half (52.9%) of the participants held a diploma, whereas the smallest percentage had an educational level of primary school or less (0.3%). The highest-reported stage of pregnancy was the third trimester (52.6%), as can be seen in Figure 1. The number of pregnancies demonstrated that 69.6% of the women were multigravida (Table 1).

Regarding plaque awareness, no association was noted between plaque awareness and age. Moreover, 32.7% of the participants answered correctly, with the highest level of awareness found in the group over 35 years, at 33%. Awareness regarding the effects of plaque was low, as only 25.6% of the participants answered correctly. Most of the study participants (85.3%) correctly answered questions about gum bleeding and what it indicates. Regarding periodontal disease prevention, most participants (87.2%) answered correctly. Awareness levels ranged from 25.7% to 92% across the 26–35 age groups (Table 2).

An association (*p* < 0.05) was noted for four questions regarding the awareness of periodontal diseases based on educational level. The definition of plaque was correctly answered by less than one-third of the study participants (102 participants; 32.7%), resulting in a *p*-value of 0.04. The effect of plaque also had a *p*-value of 0.002, with only one-quarter of the study’s participants (80 participants; 25.6%) answering correctly. The indication of bleeding gums had a *p*-value of 0.03, with the majority of participants correctly answering the question (266 participants; 85.3%). The results for the last question, with a *p*-value of 0.00, indicated a high level of awareness regarding periodontal disease prevention (Table 3).

Regarding the awareness of periodontal diseases by trimester, an association (*p* < 0.05) was observed for three of the asked questions, as only the periodontal disease prevention question did not exhibit an association (*p* = 0.11) (Table 4).

Regarding periodontal disease and general health during pregnancy, the participants were asked four questions. The first question was about the cause of inflamed gums in pregnant women, where less than half (45.2%) of the study’s participants answered with “I don’t know”, and another incorrect answer (hormonal changes) was chosen by 6.1% of the participants (Table 5).

According to the results, fear was the main reason for women not visiting dental clinics during pregnancy (11.2%). However, 13.8% of the respondents indicated that they did not see a need to make a visit. In addition, expensiveness was cited as a reason for not visiting a dental clinic by 8% of pregnant women. The pregnant women reported several reasons for not visiting the dental clinic, including fear, no perceived need to do so, and cost, with 0.6% citing these factors. The combination of feeling there was no need for dental visits and the expense associated with them was reported by 0.03%. Fear and expensiveness were reported by 6.4%. Finally, the combination of fear and no perceived need to visit the dental clinic was reported by only 1% of the respondents.

The omnibus test of the model coefficients was used to assess model fit. The results indicated significance (*p*-value < 0.05). Furthermore, the Hosmer and Lemeshow tests were not significant, implying that our model fits the data well since there is no difference between the observed and predicted models. Furthermore, the contingency table for the Hosmer and Lemeshow tests demonstrates that both values were almost equal. According to the pseudo-R-square measures, Cox and Snell (0.080) and Nagelkerke’s (0.108), the model accounts for 8.0% to 10.8% of the variation and represents a rather small effect. A 10.8% change in the criterion variables can be attributed to the predictor variables in this model.

The classification table demonstrates that the model can predict the proper category once the predictors are introduced. The model accurately classified 65.4% of cases overall based on the percentage accuracy of classification. The specification in this model, referred to as the true negative rate, indicates that 28.6% of cases observed fall into the non-targeted category. Furthermore, the sensitivity test, also known as the true positive rate, refers to the percentage of cases observed to fall into the targeted group, which is 90.3%. Overall, the accuracy rate was excellent at 65.4%. As a result, this model accurately predicted that 90.3% of pregnant women would choose yes over no.

Table 6 illustrates the logistic regression results to identify the predictors of dental visits among pregnant women. The regression model included information on the participants’ education level, pregnancy stage, age, and the number of pregnancies after fulfillment of the assumptions. In this model, pregnant women with secondary education were statistically significant and three times more likely to visit dental clinics than those with diploma degrees [AOR = 3.1; 95% Cl (1.885–7.364)]. Those aged 25 or younger were more likely to visit a dental clinic than pregnant women aged 26 or older [AOR = 1.03; 95% Cl (0.38–2.78)]. Our findings show that pregnant women during the second trimester were considered statistically significant in this model even when other variables were excluded (*p*-value = 0.015). However, when other variables were taken into consideration, the significance was reduced (*p*-value = 0.069) (Table 6).

Our findings show that the number of pregnancies plays a significant role in predicting dental visits among pregnant women. Multigravida women were 1.4 times more likely to visit a dental clinic than primigravida women [AOR = 1.43; 95% Cl (0.79–2.58)]. Furthermore, as age increases, it becomes less likely for women during pregnancy to visit dental clinics [AOR = 0.881; 95% Cl (0.38–2.78)], [AOR = 0.606; 95% Cl (0.332–1.105)]. Nonetheless, pregnant women become less likely to visit the dental clinic during their first and second trimesters [AOR = 0.884; 95% Cl (0.470–1.665)], [AOR = 0.586; 95% Cl (0.330–1.043)].

## 4. Discussion

This study demonstrated a knowledge gap among pregnant women regarding the detrimental effects of periodontal diseases. Interestingly, most participants lacked awareness of the potential association between periodontium disease and adverse pregnancy outcomes, including a low birth weight and preterm delivery. This lack of understanding extends to essential oral hygiene practices, as reflected in the low proportion (less than one-third) of the participants who accurately defined plaque and its consequences.

Our findings are in line with the observations by Alrumayh and Alfuhaid [41], further solidifying the widespread unawareness of the detrimental influence of periodontium disease on well-being during pregnancy. However, our study took a deeper dive and revealed that uncovering fear and perceived lack of need were the primary barriers to dental care utilization among nearly half of the participants who avoided clinics. Notably, previous research by de Albuquerque, Abegg, and Rodrigues [42] and Rocha and Arima [43] highlighted the fear of pain and widespread myths regarding dental treatment safety during pregnancy, which are significant factors in preventing women from seeking necessary care. In this context, healthcare professionals sometimes perpetuate these fears, as revealed by Codato and Nakama [44]. This highlights the urgent need for comprehensive education and training for healthcare providers on debunking misinformation and addressing the concerns of patients.

As reported by Togoo and Al-Almai [45] and Penmetsa and Meghana [46], pregnant women have a limited understanding of the implications of periodontium disease. Women tend to be unaware of the importance of regular dental clinic visits during pregnancy. In terms of hypothesis testing, the data show that pregnant women in the first trimester are more likely to visit dental clinics than pregnant women in the second trimester. As a result, we accepted the null hypothesis that pregnant women in their second trimester are no more likely to attend a dental clinic than those in other trimesters. Additionally, Sajjan and Pattanshetti [47] reported that pregnant women have a lower understanding of daily oral hygiene habits.

Awareness of oral diseases was found to be low in some nations, including Italy [27], Sri Lanka [28], and India [29]. This is similar to our study since there is a limited understanding of the link between low birth weight, preterm births, and periodontal conditions. Furthermore, this study is consistent with Bokhari and Sanikommu [30], who found a low awareness level in different regions of Saudi Arabia. Notably, this study found that the majority of pregnant women did not seek dental care during their pregnancy, which is consistent with a previous study conducted in the same location, which found that women often do not perceive a need to visit a dental clinic [48].

Surprisingly, our findings indicated that pregnant women with secondary and primary education levels were three times more likely to visit dental clinics compared to those with a diploma educational level. According to the literature, socioeconomic characteristics can be a strong predictor for better oral health [49]. Furthermore, a systematic analysis discovered that women with better knowledge regarding oral health during pregnancy were considerably more likely to receive dental care [27]. Overall, many research investigations indicate that a higher level of education is frequently connected with the improvement of health outcomes and greater utilization of healthcare services [50].

Our findings regarding the relationship between awareness and education level differ from those of previous research. While Bushehab and Sreedharan [37] reported no significant associations, our study demonstrated a positive link between education levels. Additionally, our results differ from those reported by Al Raeesi and Al Matrooshi [51], who observed a low utilization of dental services among pregnant women in the UAE and attributed it to limited awareness of the impact of periodontium disease during pregnancy. While the knowledge of periodontal health among pregnant Saudi Arabian women in our study was similarly inadequate, nearly half of the respondents reported seeking dental care. However, a concerning gap in awareness still exists, as most women remain unaware of the potential association between periodontal disease and adverse pregnancy outcomes like preterm births and low birth weights.

Despite this knowledge, pregnant women were found to lack knowledge regarding the role of maintaining oral health habits in addition to solving dental problems during pregnancy [52,53,54]. This lack of knowledge could be due to a number of factors. One possible cause is the misconception that dental care during pregnancy is risky or unnecessary. Another cause could be the accessibility of dental services for women during pregnancy [55,56].

Several studies with different designs, conducted within the country and abroad, have highlighted the urgent need to improve awareness among pregnant women [57,58,59]. For instance, a study in Poland revealed that barriers to awareness include socioeconomic status, education level, and dental service availability [57]. Comparably, a study in India revealed that education level is one of the barriers to awareness about oral health during pregnancy [60]. However, pregnant women in Nigeria face significant barriers to awareness due to social class [61].

This study has some limitations. As such, our findings must be interpreted with caution. First, the participants were recruited from a secondary hospital in Saudi Arabia; thus, the findings cannot be generalized to the entire population of pregnant women in Saudi Arabia. Second, our research was cross-sectional and did not assess behavior over time; as such, it only evaluated the awareness level of pregnant women. Third, this study only presented statistical significance rather than considering clinical significance. Fourth, health-related factors such as nutrition, smoking, and alcohol consumption were not considered in our exclusion criteria. As such, a cohort study design with stricter inclusion and exclusion criteria is required in future studies. Notwithstanding these limitations, this study presents a critical assessment of the awareness of pregnant women regarding the effects of periodontal conditions and pinpoints areas where knowledge is lacking.

Furthermore, this study is the first to be carried out among the Ministry of Defense’s Health Services hospitals and addresses the current gaps in the literature. Future studies should examine the necessity of evaluating the health effects of pregnancy along with their clinical significance to reduce the risk of preterm births and low birth weights caused by various diseases. Furthermore, future research should focus on developing a composite knowledge score for the overall severity of periodontal disease.

## 5. Conclusions

Pregnant women demonstrate a low level of awareness regarding the effects of periodontal disease. One-third of the surveyed women had not visited a dental clinic during their pregnancy. Several barriers to oral health awareness during pregnancy were observed, such as socioeconomic status, educational level, and routine dental clinic visits. Therefore, the best ways to enhance the health of pregnant women and newborns are through dental education clinics and campaigns aimed at raising awareness. Improvements in terms of education, promoting routine dental visits by pregnant women, and improving the socioeconomic status of the population can enhance oral health during pregnancy, which could improve overall health.

The pregnant women surveyed experienced high levels of anxiety and sensory sensitivity concerning dental visits. Addressing these concerns by expanding access to dental care and reducing anxiety about treatment outcomes can strengthen the relationship between oral health and general health.

## Figures and Tables

**Table 1:** Demographic data of the pregnant women.

Variable		Frequency	(%)
Educational level	University or master’s degree	90	(28.8)
	Secondary	56	(17.9)
	Diploma	165	(52.9)
	Primary or less	1	(0.3)
	Total	312	(100)
Age	Equal or less than 25 years	25	(8.0)
	26–30 years	77	(24.7)
	31–35 years	101	(32.4)
	More than 35 years	109	(34.9)
	Total	312	(100)
Number of pregnancies	Multigravida	217	(69.6)
	Primigravida	95	(30.4)
	Total	312	(100)

% = percentage.

**Table 2:** Questions on the awareness of periodontal disease by age.

Questions	Answers	≤25 Years	26–30 Years	31–35 Years	>35 Years	Total	*p*-Value
		N (%)	N (%)	N (%)	N (%)	N (%)	
What is plaque?	a. Correct (soft deposit)	9 (36)	24 (31.2)	33 (32.7)	36 (33)	102 (32.7)	0.223
	b. Incorrect (hard deposition on teeth, I do not know, staining)	16 (64)	53 (68.8)	68 (67.3)	73 (67)	210 (67.3)	
What can plaque cause?	a. Correct (gum disease)	8 (32)	23 (29.9)	26 (25.7)	23 (21.1)	80 (25.6)	0.132
	b. Incorrect (discoloration, malformation, I do not know)	17 (68)	54 (70.1)	75 (74.3)	86 (78.9)	232 (74.4)	
What does bleeding gum indicate?	a. Correct (gum inflammation)	21 (84)	67 (87)	85 (84.2)	93 (85.3)	266 (85.3)	0.188
	b. Incorrect ( I do not know, healthy gum)	4 (16)	10 (13)	16 (15.8)	16 (14.7)	46 (14.7)	
How can you prevent gum disease?	a. Correct (brushing and flossing)	22 (88)	71 (92)	85 (84)	94 (86)	272 (87.2)	0.233
	b. Incorrect (taking vitamin C, I don’t know, soft diet)	3 (12)	6 (8)	16 (16)	15 (14)	40 (12.8)	

N = frequency; % = percentage.

**Table 3:** Questions on the awareness of periodontal disease by education level.

Questions	Answers	University or Master’s	Secondary	Diploma	Primary or Less	Total	*p*-Value
		N (%)	N (%)	N (%)	N (%)	N (%)	
What is plaque?	a. Correct (soft deposit)	35 (38.9)	11 (19.6)	56 (34)	0 (0)	102 (32.7)	0.043 *
	b. Incorrect (hard deposition on teeth, I do not know, staining)	55 (61.1)	45 (80.4)	109 (66)	1 (100)	210 (67.3)	
What can plaque cause?	a. Correct (gum disease)	31 (34.4)	5 (8.9)	44 (28.2)	0 (0)	80 (25.6)	0.002 *
	b. Incorrect (discoloration, malformation, I do not know)	59 (65.6)	51 (91.1)	112 (71.8)	1 (100)	232 (74.4)	
What does bleeding gum indicate?	a. Correct (gum inflammation)	81 (90)	43 (76.8)	141 (85.5)	1 (100)	266 (85.3)	0.035 *
	b. Incorrect (I do not know, healthy gum)	9 (10)	13 (23.2)	24 (14.5)	0 (0)	46 (14.7)	
How can you prevent gum disease?	a. Correct (brushing and flossing)	86 (95.6)	38 (67.9)	148 (89.7)	0 (0)	272 (87.2)	0.000 *
	b. Incorrect (taking vitamin C, I don’t know, soft diet)	4 (4.4)	18 (32.1)	17 (10.3)	1 (100)	40 (12.8)	

* The statistical significance level was set at ≤ 0.05. N = frequency; % = percentage.

**Table 4:** Questions on the awareness of periodontal diseases by trimester.

Questions	Answers	First Trimester	Second Trimester	Third Trimester	Total	*p*-Value
		N (%)	N (%)	N (%)	N (%)	
What is plaque?	a. Correct (soft deposit)	23 (37.1)	36 (41.9)	43 (26.2)	102 (32.7)	0.077
	b. Incorrect (hard deposition on teeth, I do not know, staining)	39 (62.9)	50 (58.1)	121 (73.8)	210 (67.3)	
What can plaque cause?	a. Correct (gum disease)	17 (27.4)	30 (34.9)	33 (20.1)	80 (25.6)	0.008 *
	b. Incorrect (discoloration, malformation, I do not know)	45 (72.6)	56 (65.1)	131 (79.9)	232 (74.4)	
What does bleeding gum indicate?	a. Correct (gum inflammation)	51 (82.3)	73 (84.8)	142 (86.6)	266 (85.3)	0.067
	b. Incorrect (I do not know, healthy gum)	11 (17.7)	13 (15.1)	22 (13.4)	46 (14.7)	
How can you prevent gum disease?	a. Correct (brushing and flossing)	55 (88.7)	76 (88.4)	141 (86)	272 (87.2)	0.119
	b. Incorrect (taking vitamin C, I don’t know, soft diet)	7 (11.3)	10 (11.6)	23 (14)	40 (12.8)	

* The statistical significance level was set at ≤ 0.05. N = frequency; % = percentage.

**Table 5:** Awareness regarding periodontal disease and general health.

Questions	Answers	Frequency	(%)
What causes gum inflammation in pregnant women?	a. Neglecting brushing	83	(26.6)
	b. Dental plaque	55	(17.6)
	c. Hormonal changes	19	(6.1)
	d. I do not know	141	(45.2)
	e. Plaque and neglecting	14	(4.5)
	Total	312	(100.0)
Do you think tooth brushing should be increased during pregnancy?	a. Yes	240	(76.9)
	b. No	35	(11.2)
	c. I do not know	37	(11.9)
	Total	312	(100.0)
Do you think that gum disease would lead to the delivery of a preterm or low-birth-weight infant?	a. Yes	92	(29.5)
	b. No	163	(52.2)
	c. I do not know	57	(18.3)
	Total	312	(100.0)
Do you think that smoking has a negative effect on the pregnant woman and her child?	a. Yes	303	(97.1)
	b. No	7	(2.2)
	c. I do not know	2	(0.6)
	Total	312	(100.0)

% = percentage.

**Table 6:** Binary logistic regression model to predict dental visits among pregnant women.

Variables	*p*-Value	AOR (95% Cl)	*p*-Value	COR (95% Cl)
**Education level**				
Secondary or primary	0.002 *	3.1.36 (1.885–7.364)	0.002 *	3.047 (1.527–6.081)
Diploma	0.539	0.842 (0.486–1.458)	0.477	0.842 (0.482–1.406)
University level (Ref.)		1.0		1.0
**Age**				
Less than or equal to 25 years	0.949	1.033 (0.383–2.782)	0.595	0.787 (0.325–1.905)
26–30 years	0.705	0.881(0.456–1.700)	0.196	0.674 (0.371–1.226)
31–35 years	0.102	0.606 (0.332–1.105)	0.227	0.712 (0.410–1.235)
More than 35 years (Ref.)		1.0		1.0
**Number of pregnancies**				
Primigravida	0.237	0.700 (0.387–1.265)	0.112	0.665 (402–1.099)
Multigravida (Ref.)		1.0		1.0
**Stage of pregnancy**				
First trimester	0.703	0.884 (0.470–1.665)	0.304	0.731 (0.403–1.327)
Second trimester	0.069	0.586 (0.330–1.043)	0.015 *	0.502 (0.289–0.872)
Third trimester (Ref.)		1.0		1.0

Cl = confidence level; AOR = adjusted odds ratio; COR = crude odds ratio; * = significant *p*-value level at ≤ 0.05.

## Data Availability

The original contributions presented in the study are included in the article/supplementary material; further inquiries can be directed to the corresponding author.

## Supplementary Materials

The following supporting information can be downloaded at: https://www.mdpi.com/article/10.3390/healthcare12232413/s1, The Questionnaire.
