# Supplementary material for: Pregnant Women’s Awareness of Periodontal Disease Effects: A Cross-Sectional Questionnaire Study in Saudi Arabia
Source: Healthcare (Basel). 2024 Dec 1;12(23):2413. doi: 10.3390/healthcare12232413 (PMC11641118; doi:10.3390/healthcare12232413)
Supplement: Supplementary file 1 [file healthcare-12-02413-s001.zip › healthcare-3328183-supplementary.pdf]

The Questionnaire.

**1. Age**<br>☐ ⩽ 25 ☐ 26-30 ☐ 31-35 ☐ >35	1.العمر<br>☐ ⩽ 25 ☐ 26-30 ☐ 31-35 ☐ >35
**2. Educational Level:**<br>☐ Primary or less ☐ Secondary ☐ Diploma ☐ University or master degree	2. المستوى العلمي:<br>☐ ابتدائي أو اقل ☐ المرحلة الثانوية ☐ دبلوم<br>☐ بكالوريوس جا□عة أو دراسات عليا
**3. Stage of pregnancy:**<br>☐ First trimester ☐ Second trimester ☐ Third trimester	3.□رحلة الحمل الحالية:<br>☐ الثلاثة شهور الأولى ☐ الثلاثة شهور<br>الثانية ☐ الثلاثة شهور الأخيرة
**4. Number of pregnancies:**<br>☐ Primigravidae ☐ Multigravidae	4. الحمل الحالي هو:<br>☐ الأول<br>☐ الثاني أو أكثر
**5. What is plaque?**<br>☐ Soft deposition on teeth ☐ Hard deposition on teeth ☐ Staining ☐ I don't know	5.□ا هي الصفيحة الجرثو□ية (البلاك) ؟<br>☐ رواسب ناعمة على الأسنان ☐<br>□صبغ على □رواسب قاسية على الأسنان<br>لا اعلم□الأسنان
**6. What can plaque cause?**<br>☐ Discoloration of teeth ☐ malformation ☐ Gum disease ☐ I don't know	6.□ا□أثيرها على الأسنان؟<br>☐ اختلاف لون الأسنان ☐□شوه الأسنان<br>☐□شاكل في اللثة ☐ لا اعلم
**7. Bleeding gum indicates:**<br>☐ Gum inflammation ☐ Gum recession ☐ Healthy gum ☐ I don't know	7. نزيف اللثة يدل على:<br>انحسار في ☐ التهاب اللثة ☐<br>لا اعلم ☐ لثة سليمة ☐اللثة
**8. Gum disease could be prevented through:**<br>☐ Brushing& flossing ☐ soft diet ☐ By taking Vitamin C ☐ I don't know	8. يمكن الحد□ن أ□راض اللثة عن طريق:<br>□فريش الأسنان و استعمال خيط الاسنان ☐<br>□ناول فيتا□ين ☐□ناول الاطعمة غير القاسية ☐<br>لا اعلم☐سي
**9. The most important time to brush teeth is:**<br>☐ In the morning ☐ Midday ☐ Before sleeping ☐ No brushing ☐ more than 1 time	9. أفضل وقت لتفريش الأسنان هو:<br>☐ في الصباح ☐ في□نتصف اليوم ☐ قبل<br>الخلود إلى النوم ☐ لا داعي للتفريش ☐أكثر<br>□ن□رة
**10. What causes inflamed gum in pregnant women?**<br>☐ Dental plaque ☐ Hormonal changes ☐ Neglecting brushing ☐Don't know ☐Plaque and neglecting	10.□ا هي□سببات التهاب اللثة لدى المرأة<br>□لحا□ل؟<br>□غير ☐ الصفيحة الجرثو□ية(البلاك) ☐<br>إهمال□فريش الأسنان ☐الهر□ونات في الجسم<br>الصفيحة الجرثو□ية (البلاك) و ☐لا اعلم ☐<br>الأهمال
**11. Do you think tooth brushing should be increased during pregnancy?**<br>☐ Yes ☐ No ☐ I don't know	11. هل□عتقدين بوجوب زيادة□فريش الأسنان<br>خلال فترة الحمل؟<br>لا ☐ نعم ☐<br>لا اعلم☐

**12. Do you think that gum disease would lead to the delivery of a preterm or low-birth weight infant?**<br>☐ Yes ☐ No ☐ I don't know	12. هل□عتقدين أن□رض اللثة□مكن أن يؤدي إلى إنجاب طفل□بكرا و بوزن قليل؟<br>لا □ نعم □<br>لا اعلم□
**13. Do you think that smoking has a negative effect on the pregnant woman and her child?**<br>☐ Yes ☐ No ☐ I don't know	13. هل□عتقدين أن التدخين له□أثير سلبي على المرأة الحا□ل و طفلها؟<br>لا □ نعم □<br>لا اعلم□
**14. The bristles of your toothbrush are**:<br>☐ Soft ☐ Hard ☐ I don't brush my teeth	14. نوع فرشاة الأسنان التي□ستخد□ينها:<br>لا افرش □ خشنة □ناعمة □<br>أسناني
**15. do you visit your dentist regularly :**<br>☐ **Yes** ☐No	15.هل□زورين طبيب الأسنان بانتظام:<br>لا□نعم □
**16. If no, then the reason is:**<br>☐ Fear ☐ No need to do so ☐ Expensive	16. إذا أجبتي بلا فالسبب هو:<br>لا ضرورة لذلك□ الخوف □<br>ار□فاع□كاليف العلاج□
